# Segmented linear correlations between bone scan index and prostate cancer biomarkers, alkaline phosphatase, and prostate specific antigen in patients with a Gleason score ≥7

**DOI:** 10.1097/MD.0000000000029515

**Published:** 2022-06-24

**Authors:** Ebrahim Tasmeera, Hadebe Bawinile, Aldous Colleen, Partson Tinarwo, Nozipho Nyakale

**Affiliations:** a Department of Nuclear Medicine, College of Health Sciences, University of KwaZulu-Natal, Durban, South Africa; b Department of Nuclear Medicine, Inkosi Albert Luthuli Central Hospital, Durban, South Africa; c College of Health Sciences, School of Medicine, University of KwaZulu-Natal, Durban, South Africa; d Department of Biostatistics, Nelson Mandela School of Medicine, University of KwaZulu-Natal, Durban, South Africa.; e Department of Nuclear Medicine, Sefako Makgatho Health Sciences University and Dr George Mukhari Academic Hospital, Pretoria, South Africa.

**Keywords:** alkaline phosphatase, bone scan index, bone scan, prostate cancer, prostate specific antigen

## Abstract

Technetium-99m methyl diphosphonate bone scintigraphy is relatively easily accessible for detecting bone metastases in prostate cancer patients. However, it is subjective and can be challenging to compare images taken at different time points. The bone scan index (BSI) is a more objective evaluation and allows for better comparison of images. Its correlation with other biomarkers of prostate cancer metastases such as prostate specific antigen (PSA) and alkaline phosphatase (ALP) is not clearly understood. This study thus aimed to compare the BSI correlation to PSA against that of BSI to ALP levels in patients with a Gleason score ≥7.

A retrospective analysis of the medical records of 50 prostate cancer patients with a Gleason score of ≥7 referred for a bone scan between January 1, 2015 and December 31, 2018 was undertaken. Bone scans were interpreted visually, and using a semi-automated computer programme to quantify the BSI and its relation to PSA and ALP measurements.

For the metastasis positive measurements, there was a statistically significant moderate positive overall linear correlation between BSI and PSA. For ALP and BSI, there were 2 segmented strong positive linear relationships between them. The first segment consisted of ALP < 375 IU/L and BSI >10%, where ALP and BSI were strongly and positively correlated. The other segment tended to have generally low BSI measurements (<10%) and also had a strong and positive correlation.

The BSI was found to be better linearly correlated with ALP than PSA.

## 1 Introduction

The prevalence of prostate cancer has been on the rise in recent years.^[1]^ A common site of metastasis from prostate cancer is bone. Detecting bone metastases is important in assessing prognosis as well as in identifying and preventing complications that may occur as a result of disease progression. Bone scintigraphy, using Technetium-99m methyl diphosphonate (Tc-99m MDP), is an excellent modality for detecting bone metastases in that it is highly sensitive, relatively easily accessible, non-invasive, and allows one to assess the entire skeleton. According to a systematic review and meta-analysis carried out by Zhou et al,^[2]^ the per-patient pooled sensitivity of bone scintigraphy in prostate cancer patients was 0.86. In another meta-analysis undertaken by Shen et al,^[3]^ they demonstrated that the pooled sensitivity of bone scintigraphy, on a per-patient basis, in prostate cancer patients was 0.79. Bone scintigraphy; however, is a subjective evaluation, and it is difficult to draw comparisons of images taken over a period of time using this modality alone. The bone scan index (BSI) offers a more objective evaluation and further allows for quantification of the bone scan findings.

Prostate specific antigen (PSA) levels are often used as an indication for the presence of bone metastases and have been used as a gate-keeper to determine the need for bone imaging. A study carried out by Johnston et al^[4]^ showed that a higher PSA at scan was significantly predictive of bone scan positivity (*P* < .0001). Various studies have also assessed the value of alkaline phosphatase (ALP) in the prediction of bone metastases and suggested that ALP may be the more reliable indicator when stratifying the extent of bone metastases. According to Nakajima et al,^[5]^ the BSI closely correlates with bone alkaline phosphatase, while its correlation with PSA is only fair. However, Imbriaco et al,^[6]^ have demonstrated that the changes in BSI correlated with the changes in PSA during disease progression. Most of the studies done to date have independently compared PSA with BSI or ALP with BSI. This study is unique because it is a direct comparison of PSA and ALP to BSI to assess disease burden, using BSI as the gold standard.

Although helpful in detecting tumor and showing progression, PSA levels do not correlate in absolute terms with the tumor burden.^[6]^ For a given tumor size, the PSA value varies widely from patient to patient.^[6]^ More poorly differentiated tumors produce less PSA per gram than do well-differentiated tumors, and serum levels in a given patient may also be influenced by the amount of benign prostatic hypertrophy in residual prostate tissue.^[6]^ Also, for more advanced tumors, PSA levels can be increased both by soft tissue and bony metastases; therefore, metastatic disease to bone is not monitored directly.^[6]^

ALP, one of the older biochemical tools for investigating and monitoring prostate cancer, has stood the test of time and remains a reliable indicator of osteoblastic activity, as in bone metastases.^[7]^ The univariate and multivariate regression analyses carried out by Chaoying et al^[8]^ in their study confirmed that ALP was an independent risk factor and predictor of bone metastases in prostate cancer. According to Akimoto et al,^[9]^ PSA was inferior to ALP for stratifying the metastatic burden of bone. They demonstrated that PSA was the best marker for differentiating clinical stages but showed limited reliability for stratifying the extent of bone metastases.^[9]^ Adding ALP determination improved the evaluation of the stratification.^[9]^

A recent publication by Yordanova et al^[10]^ highlights the value of both s-PSA and ALP as tumor markers in the management of prostate cancer. The authors demonstrated a strong correlation between the kinetic patterns of both tumor markers with patient survival. Baseline ALP and PSA correlated significantly with overall survival (OS) and demonstrated similar trends on patient survival analysis. A 2018 meta-analysis on the prognostic value of ALP in prostate cancer patients included 63 studies with a total of 16,135 patients. Pooled results demonstrated that a high serum PSA was significantly associated with a poor OS and progression-free survival (in a manner similar to PSA).^[11]^ Heinrich et al^[12]^ also recently reassessed the current role and possible value of ALP in patients with metastatic castrate-resistant prostate cancer in the setting of various available treatment modalities. In patients treated with Docetaxel, ALP could be used to differentiate PSA flare from early PSA progression. The authors concluded that ALP would be a reasonable prognostic marker (especially with regards to OS) for the routine monitoring in patients with bone-dominant metastatic castrate-resistant prostate cancer, regardless of the treatment modality used.

The BSI was developed as a fully quantifiable estimation of the osseous disease tumor burden.^[13]^ This index represents the fraction of the skeletal mass with abnormal tracer uptake indicative of metastatic disease on a bone scan.^[13]^ In addition to quantifying metastatic skeletal involvement as well as therapeutic response, the BSI is also useful for prognostic purposes.^[5]^ A study of patients with castration-resistant prostate cancer found that a doubled BSI resulted in a 1.9-fold increase in death risk.^[5]^ BSI can also be used to risk-stratify patients, as illustrated by the study carried out by Kaboteh et al.^[14]^ The study showed that patients with metastatic disease and BSI < 1 showed a 5-year probability of survival of 42% compared with 31% for those with BSI 1–5 and 0% for those with BSI >5.^[14]^

Our study aimed to determine the role of PSA and ALP as prostate cancer biomarkers by comparing the BSI correlation to PSA against that of BSI correlation to ALP levels in patients with a Gleason score ≥7. The Gleason grading system developed by Dr. Donald Gleason in 1966, remains the cornerstone for the management of prostate cancer.^[15]^ The system is relatively simple and reasonably reproducible to apply.^[15]^ It is one of the key parameters for therapy-planning (active surveillance vs definitive therapy), and remains as the most important prognostic factor in predicting pathological findings in radical prostatectomy, biochemical failure, local and distant metastasis after therapy, and prostate cancer specific mortality.^[15]^ Five cellular architectural patterns observed in prostatic tissue are characterized: 1, 2, and 3 representing normal prostate tissue, and 4 and 5 indicative of cancer or abnormal tissue.^[16]^ The score is the sum of the 2 most common patterns observed in tumor samples.^[16]^

The most widely used classification to risk-stratify prostate cancer patients is the D’Amico classification, which divides patients into low-, intermediate-, and high-risk groups^[17]^ and takes into consideration PSA level, Gleason score, and clinical tumor stage. A Gleason score of ≤6 falls into the low-risk group, 7 intermediate-risk group, and 8 to 10 high-risk group. Bone metastases are seen in 5% of patients with a Gleason score of <6 versus 30% of those with a Gleason score of >7.^[17]^ According to Donohoe et al,^[17]^ bone scintigraphy is usually appropriate for initial staging in patients with intermediate- and high-risk disease, and usually not appropriate for initial staging in patients with a low risk of metastatic disease. Based on this, patients with a Gleason score of ≥7 were included in our study population as they had a higher likelihood of having bone metastases.

## 2 Methods

Ethical approval was obtained from our institution's Biomedical Research Ethics Committee (reference number BREC/00001014/2020).

### 2.1 Study population

The study was undertaken at the Department of Nuclear Medicine, where bone scans of prostate cancer patients carried out from January 01, 2015 to December 31, 2018 were evaluated. Prostate cancer patients with a Gleason score ≥7 with documented PSA and ALP results were selected. Patients who have had a bone scan done in the study period with no PSA or ALP results available, as well as those with known hyperparathyroidism and pre-existing bone diseases, were excluded. For patients who had more than one bone scan in the study period, only the initial bone scan was evaluated in this study. The final study group therefore consisted of 50 bone scans (median age of the patients was 65.5 [63.0–72.8] years old and ranged from 52 to 83 years old).

### 2.2 Bone scintigraphy procedure

All patients were injected with 20 mCi of Tc-99m MDP intravenously, followed by whole-body anterior and posterior planar imaging at 2.5 hours post-injection using a Siemens Symbia gamma camera equipped with a low-energy high-resolution parallel hole collimator. The camera parameters were as follows: scan speed of 10 cm/min, matrix size of 256 × 1024 and energy window set at 15% over 140 keV photopeak.

### 2.3 Image analysis

Each bone scan was assessed by the principal investigator and an experienced Nuclear Physician to evaluate for metastatic disease. If there was any discrepancy in their findings, a third independent Nuclear Physician assessed the bone scan concerned to reach a consensus. Lesions were considered metastatic depending on the pattern of uptake seen. Multiple foci of increased radiotracer uptake randomly distributed in the skeleton were considered metastatic lesions. Diffusely increased uptake seen in the skeleton with faint or absent visualization of the kidneys, which is in keeping with a “superscan,” was considered indicative of diffuse metastases being present. Solitary lesions were considered metastatic depending on the location—lesions in the central skeleton have a higher likelihood of being metastatic and were thus considered metastatic in our study.

Areas of increased radiotracer uptake were considered negative for metastasis if they: localized to joints (likely indicative of inflammatory or degenerative change), appeared to be due to urinary contamination, or localized to areas of known trauma or the pattern of uptake was suggestive of trauma (e.g., focal uptake in contiguous ribs). In addition, lesions were also correlated with age, and PSA and ALP results. Although bone histology is the gold standard of diagnosing bone metastasis, it is not practical or ethical to biopsy each bone lesion present. A best valuable comparator was thus used taking into consideration all available information including follow-up bone scans, Tc-99m PSMA scans, F-18 FDG PET/CT scans, Ga-68 PSMA PET/CT scans, X-rays, CT scans as well as follow-up clinical data.

All bone scan images were saved in Digital Imaging and Communications in Medicine format. Automated BSI analysis was performed using the program developed by EXINI Diagnostics, Lund, Sweden. The programme segments the skeleton (excluding the distal arms and legs) into different anatomical regions in the anterior and posterior views. Hotspots, which are defined as areas of increased radiotracer (Tc-99m MDP) uptake in comparison to the surrounding skeleton, are then detected. These are then classified as either being metastatic lesions or not. The metastatic hotspots area is calculated and then divided by the area of the corresponding anatomical region. The result is then multiplied by a constant representing the weight fraction of the concerned skeletal region with respect to the weight of the total skeleton. This value gives an estimate of the volumetric fraction of the skeletal region occupied by the metastatic hotspots. The BSI equates to the sum of all such fractions. A BSI of 0% was classified as negative while any BSI > 0% was regarded as positive.

### 2.4 PSA and ALP results

The PSA (in ng/mL) and ALP (in IU/L) levels of the patients were determined from the hospital laboratory records or National Health Laboratory Services TrakCare website. PSA levels were correlated with BSI using the following sub-groups: <200, 200 to <1000, and ≥1000 ng/mL. ALP was classified using <375 and >375 IU/L. Optimal cut-off values of PSA and ALP for predicting the presence of metastases were determined.

### 2.5 Data analysis

The statistical data analysis was conducted using R Statistical computing software version 3.6.3. Descriptive statistics such as count and percentage frequencies were used to summarize categorical data and with the aid of an alluvial diagram for multidimensional crosstabulations. Either chi-square or Fisher exact tests were used to assess the association between categorical demographics and the metastasis findings. Due to skewness, numerical variables were summarized using the median and interquartile range. The median differences between the positive and negative metastasis measurements were assessed using non-parametric rank sum test. A parallel plot was used to multidimensionally visualize the behavioral patterns of the medians of the numerical measures within race categories. Pearson correlation coefficient or Spearman rank correlation coefficients were applied to measure the strength of the linear relationship between the BSI and the blood markers (PSA and ALP) for the metastasis positive patients. In addition, segmented linear regression equations were fitted and graphically visualized. Sensitivity and specificity analysis were conducted using the R function multi_cutpointr in the library cutpointr to determine the optimal cut-off points of PSA, ALP, and age that were likely to be indicative of metastasis positive patients based on the Youden index. The segmented correlations consisted of at least 5 data points and a post-hoc power analysis was conducted using G Power software for sample size calculation version 3.1.9.7. The minimum sample size of 5 data points was found to detect a correlation that differs from 0 by at least 0.84 about 83% of the time (power of test) with 95% confidence. All the tests were conducted at 5% significance level.

## 3 Results

The study consisted of 50 patients aged 52 to 83 years old (median 65.5 [Q1–Q3 63.0–72.8] years old). The majority, that is, 50% (25/50) of the study population were Black, 26% (13/50) were White, 22% (11/50) were Indian, and 2% (1/50) were Colored. PSA levels ranged from 0.0400 to 2790 ng/mL (median 26.8 [Q1–Q3 2.86–124] ng/mL). ALP levels ranged from 37.0 to 1490 IU/L (median 95.5 [Q1–Q3 66.5–166] IU/L). The BSI ranged from 0% to 35.20% (median 0 [Q1–Q3 0–3.40]%).

### 3.1 Association between bone metastases and demographics

Of the 50 bones scans that were assessed for metastatic disease (Table 1), results showed a prevalence of bone metastases of 44% (n = 22). Among the metastasis positive bone scans, 59.1% (13/22) were Blacks, 31.8% (7/22) were Whites, 9.1% (2/22) were Indians, and none were Colored. The overall median (Q1–Q3) age of the patients was 65.5 (63.0–72.8) years old and ranged from 52 to 83 years old. There was no statistically significant difference in the median ages between the positive and negative patients for bone metastases, (*P* = .144). Similarly, there was no association between race and the presence or absence of metastases (*P* = .163).

**Table 1:** Patient characteristics according to the metastatic findings.

	Metastases			
	Negative 56% (N = 28)	Positive 44% (N = 22)	*P*-value	Overall (N = 50)
Age			.144	
Median (Q1–Q3)	64.5 (61.8–70.3)	69.5 (64.0–73.8)		65.5 (63.0–72.8)
Min–Max	55.0–77.0	52.0–83.0		52.0–83.0
Race			.163	
Black	12 (42.9%)	13 (59.1%)		25 (50.0%)
Colored	1 (3.6%)	0 (0%)		1 (2.0%)
Indian	9 (32.1%)	2 (9.1%)		11 (22.0%)
White	6 (21.4%)	7 (31.8%)		13 (26.0%)
PSA			<.001	
Median (Q1–Q3)	5.61 (0.738–26.4)	155 (54.5–598)		26.8 (2.86–124)
Min–Max	0.0400–180	0.110–2790		0.0400–2790
ALP			<.001	
Median (Q1–Q3)	75.0 (64.0–101)	165 (107–268)		95.5 (66.5–166)
Min–Max	46.0–388	37.0–1490		37.0–1490
Bone scan index			<.001	
Median (Q1–Q3)	0 (0–0)	3.90 (1.08–14.0)		0 (0–3.40)
Min–Max	0–0	0.100–35.2		0–35.2
PSA duration			.497	
<6 months	26 (92.9%)	22 (100%)		48 (96.0%)
6+ months	2 (7.1%)	0 (0%)		2 (4.0%)
ALP duration			.522	
<6 months	14 (50.0%)	13 (59.1%)		27 (54.0%)
6+ months	14 (50.0%)	9 (40.9%)		23 (46.0%)

PSA and ALP measurements were significantly higher in patients with positive BSI compared with patients with negative BSI for bone metastasis. ALP = Alkaline phosphatase, max = maximum, min = minimum, N = number of patients, PSA = prostate specific antigen. The *P*-values are based on non-missing cases only (tableStack).

### 3.2 Association between bone metastases and PSA and ALP

The metastasis positive bone scans had a median (Q1–Q3) BSI of 3.90 (1.08–14.0)% and ranged from 0.100% to 35.2%. Both the PSA and ALP measurements were significantly higher (*P*-values < .001) in patients with positive BSI compared with patients with negative BSI for bone metastasis. The metastasis negative and positive bone scans had median (Q1–Q3) PSA measurements of 5.61 (0.738–26.4) and 155 (54.5–598) ng/mL, respectively. On the other hand, the metastasis negative and positive bone scans had median (Q1–Q3) ALP measurements of 75.0 (64.0–101) and 165 (107–268) IU/L, respectively.

### 3.3 Association between bone metastases and timing of PSA and ALP measurements

**Figure 1:**
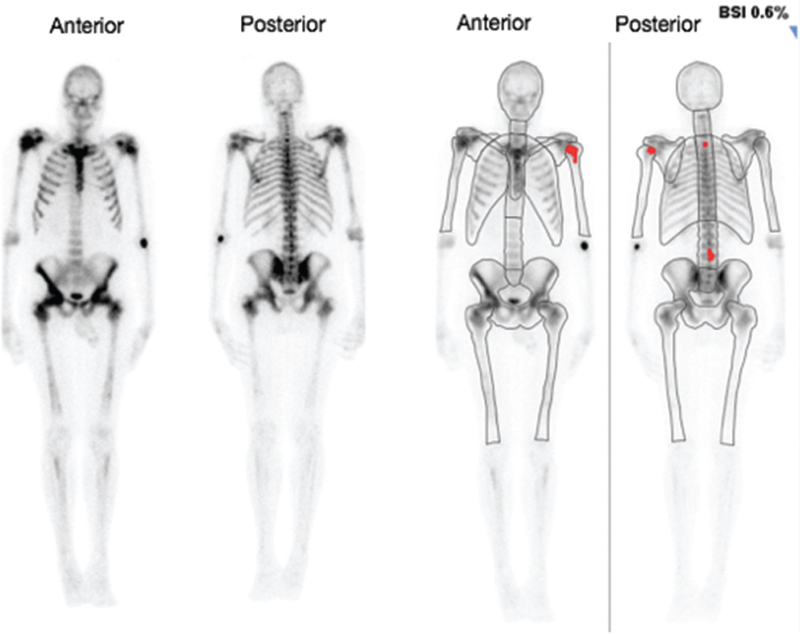
PSA and ALP durations by race among the metastasis positive patients. Of the 22 metastasis positive patients, all of them had their initial PSA measurements taken within 6 months prior to the bone scan. 59.1% of the patients had their ALP measurement within 6 months prior to the bone scan and in 40.9% of the patients it was obtained more than 6 months earlier. There was no association between race and the duration (months) of the records since they were measured (PSA [*P* = .497] and ALP [*P* = .522]). ALP = alkaline phosphatase, PSA = prostate specific antigen.

Of the 22 metastasis positive patients, all of them had their initial PSA measurements taken within 6 months prior to the bone scan, and 59.1% (13/22) of the patients also had their ALP measurement within 6 months prior to the bone scan (Fig. 1). Of the 40.9% (9/22) whose ALP measurements were obtained >6 months earlier, 18.2% (4/22) were White, 18.2% (4/22) were Black, and 4.5% (1/22) were Indian. Of note, is that there was no association between race and the duration (months) of the records since they were measured (PSA [*P* = 0.497] and ALP [*P* = 0.522]).

### 3.4 Association between BSI, PSA, and ALP with demographics

**Figure 2:**
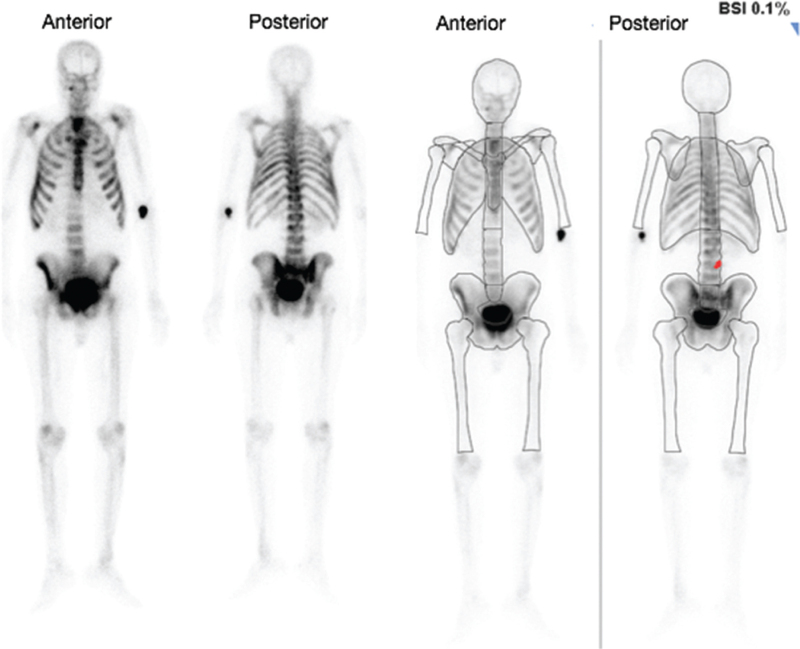
Median distribution of the biomarkers for the metastasis positive patients by race. A parallel plot of the metastasis positive measurements revealed that the elderly patients were mainly Whites (median age 70.0 years old). All the medians of BSI 2.50%, ALP 77.0 IU/L, and PSA 30.7 ng/mL were lowest in the oldest patients who were of the White ethnic group while the 2 youngest Indian patients showed the highest medians for BSI and ALP. PSA was relatively very high among Blacks when compared to the other ethnic groups. ALP = alkaline phosphatase, BSI = bone scan index, PSA = prostate specific antigen.

A parallel plot of the metastasis positive measurements revealed that the elderly patients were mainly Whites (median [Q1–Q3] age 70.0 [62.5–73.0] years old) followed by Blacks (69.0 [64.0–75.0] years old) and then Indians (68.5 [67.3–69.8] years old) (Fig. 2 and Table 2). Further, all the medians (Q1–Q3) of BSI 2.50 (0.300–9.60)%, ALP 77.0 (65.5–162) IU/L, and PSA 30.7 (15.6–324) ng/mL were lowest in the oldest patients who also happened to be of the White ethnic group. The 2 youngest Indian patients showed the highest medians for BSI and ALP. Another notable pattern was that PSA was relatively very high among Blacks with median (Q1–Q3) 350 (75.2–1 370) ng/mL as compared with the PSA medians of Indians and Whites which were under 82.0 ng/mL. However, the medians (Q1–Q3) of BSI and ALP were both relatively moderate for the Blacks, that is, 4.10 (1.70–15.7)% and 167 (138–275) IU/L, respectively, as compared with the medians for Indians (highest) and Whites (lowest).

**Table 2:** Association between BSI, PSA, and ALP with patient race including Black, White, Indian, and Colored South Africans.

Race	Black (N = 13)	Indian (N = 2)	White (N = 7)	Overall (N = 22)
Age				
Median (Q1–Q3)	69.0 (64.0–75.0)	68.5 (67.3–69.8)	70.0 (62.5–73.0)	69.5 (64.0–73.8)
Min–Max	52.0–83.0	66.0–71.0	55.0–77.0	52.0–83.0
PSA				
Median (Q1–Q3)	350 (75.2–1370)	81.6 (75.0–88.1)	30.7 (15.6–324)	155 (54.5–598)
Min–Max	22.1–2790	68.4–94.7	0.110–2080	0.110–2790
ALP				
Median (Q1–Q3)	167 (138–275)	257 (220–294)	77.0 (65.5–162)	165 (107–268)
Min–Max	103–1490	183–331	37.0–296	37.0–1490
Bone scan index				
Median (Q1–Q3)	4.10 (1.70–15.7)	4.35 (2.98–5.73)	2.50 (0.300–9.60)	3.90 (1.08–14.0)
Min–Max	0.100–35.2	1.60–7.10	0.100–15.9	0.100–35.2

ALP = alkaline phosphatase, max = maximum, min = minimum, N = number of patients, PSA = prostate specific antigen. The *P*-values are based on non-missing cases only (tableStack).

### 3.5 Correlation between BSI and PSA

**Figure 3:**
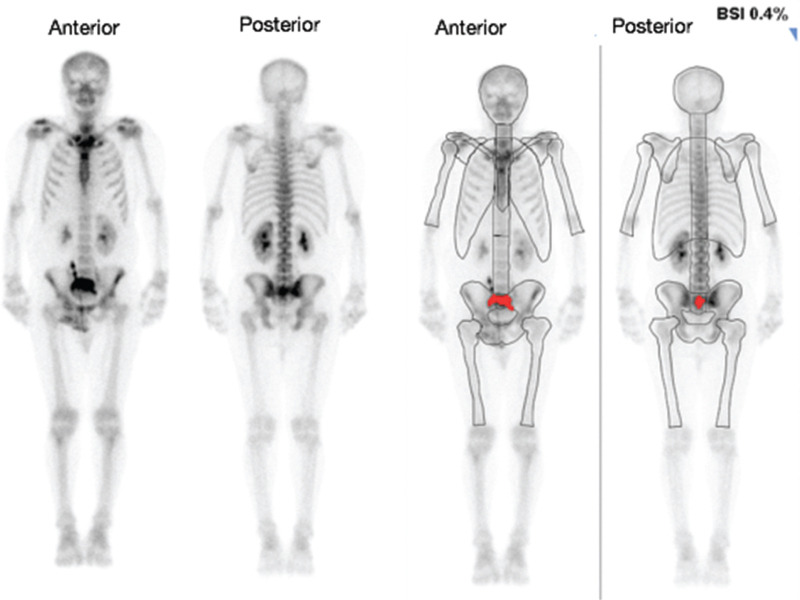
The overall correlation between BSI and PSA in metastasis positive patients. For the metastasis positive measurements, there was a statistically significant moderate positive overall linear correlation (*r* = 0.54, *P* = .009) between BSI and PSA. The associated simple linear regression equation suggested that the BSI increased by 0.005 units for an every 1 ng/mL increase in PSA. BSI = bone scan index, PSA = prostate specific antigen.

**Figure 4:**
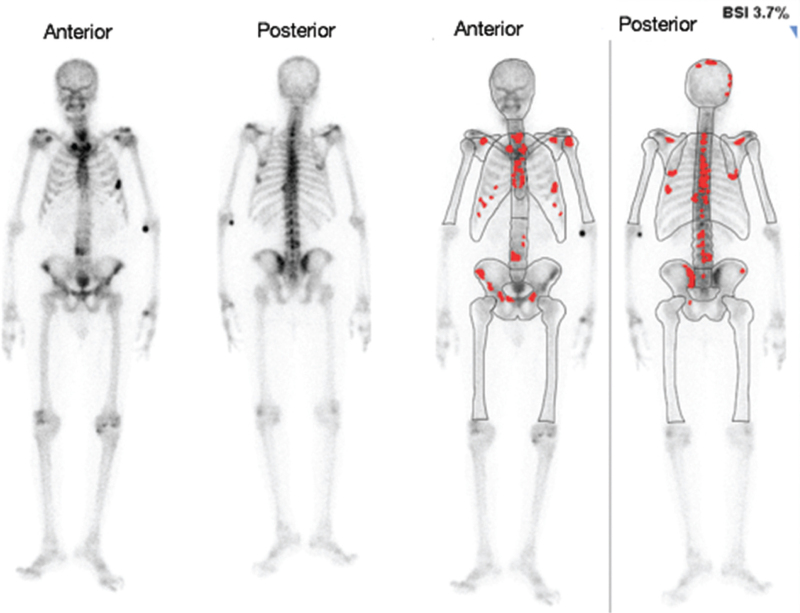
The segmented correlation between BSI and PSA in metastasis positive patients. The relationship between PSA and BSI was more defined within 3 segments that were based on PSA measurements. However, PSA and BSI positive correlations within each segment were statistically insignificant, (PSA < 200 ng/mL, *r* = 0.55, *P* = .077); (PSA 200 to <1000 ng/mL, *r* = 0.79, *P* = .064); and (PSA ≥ 1000 ng/mL, *r* = 0.80, *P* = .100). BSI = bone scan index, PSA = prostate specific antigen.

For the metastasis positive measurements, there was a statistically significant moderate positive overall linear correlation (*r* = 0.54, *P* = .009) between BSI and PSA (Fig. 3). The associated simple linear regression equation suggested that the BSI increased by 0.005 units for an every 1 ng/mL increase in PSA. Figure 4 provides an indication that the relationship between PSA and BSI was more defined within 3 segments that were based on PSA measurements. However, PSA and BSI positive correlations within each segment were statistically insignificant, (PSA < 200 ng/mL, *r* = 0.55, *P* = .077); (PSA 200 to <1000 ng/mL, *r* = 0.79, *P* = 0.064); and (PSA ≥ 1000 ng/mL, *r* = 0.80, *P* = .100).

### 3.6 Correlation between BSI and ALP

**Figure 5:**
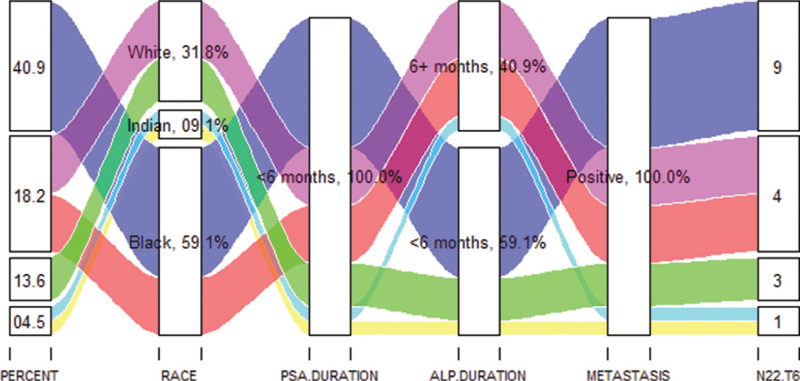
The segmented correlation between BSI and ALP in metastasis positive patients. Two segments were observed for the BSI and ALP linear relationships but the segmentation was quite complex. The first segment consisted of ALP < 375 IU/L and BSI > 10%, where ALP and BSI were strongly and positively correlated (*r* = 0.91, *P* = .029) with a unit increase in ALP found to see a significant increase in the BSI by 0.095%. The other segment tended to have generally low BSI measurements (<10%) and also had a strong and positive correlation (*r* = 0.86, *P* < .001), with the BSI increasing by 0.024% for a 1 IU/L increase in ALP. ALP = alkaline phosphatase, BSI = bone scan index.

Two segments were observed for the BSI and ALP linear relationships but the segmentation was quite complex (Fig. 5). The first segment consisted of ALP <375 IU/L and BSI > 10%, where ALP and BSI were strongly and positively correlated (*r* = 0.91, *P* = 0.029) with a unit increase in ALP found to see a significant increase in the BSI by 0.095%. The other segment tended to have generally low BSI measurements (<10%) and also had a strong and positive correlation (*r* = 0.86, *P* < .001), with the BSI increasing by 0.024% for a 1 IU/L increase in ALP.

### 3.7 Correlation between BSI and bone metastases

**Figure 6:**
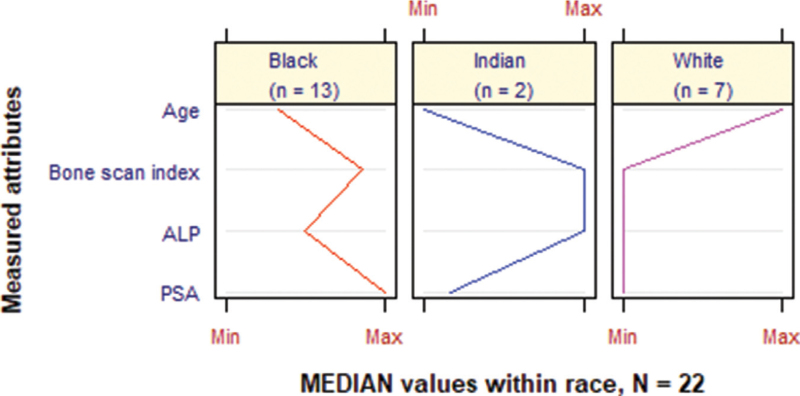
Patient 30 with a “superscan.” The anterior and posterior images to the left are the original bone scan images and the anterior and posterior images to the right are the processed BSI images. The BSI correctly identified the case as positive for bone metastases (BSI of 0.6%); however, it grossly underestimated the extent of the metastases. It only identified a few of the metastatic lesions present (as marked in red) whereas in this case the entire skeleton is diffusely involved. BSI = bone scan index.

**Figure 7:**
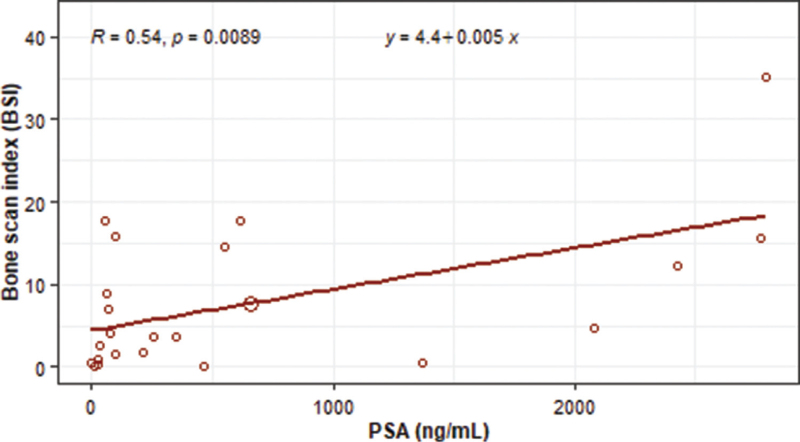
Patient 46 with a “superscan.” The anterior and posterior images to the left are the original bone scan images and the anterior and posterior images to the right are the processed BSI images. The BSI correctly identified the case as positive for bone metastases (BSI of 0.1%); however, it grossly underestimated the extent of the metastases. It only identified a single metastatic lesion present (as marked in red) whereas in this case the entire skeleton is diffusely involved. BSI = bone scan index.

**Figure 8:**
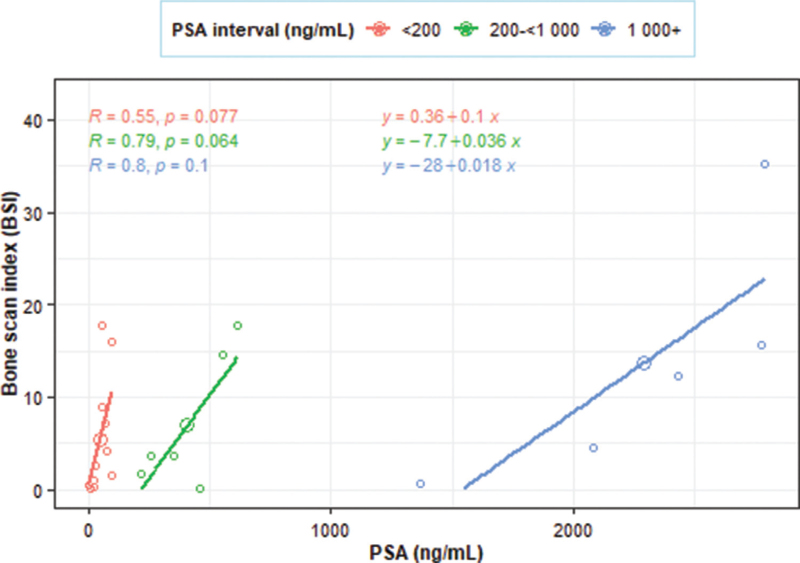
The anterior and posterior images to the left are the original bone scan images and the anterior and posterior images to the right are the processed BSI images. In this patient (patient 42), the BSI was incorrectly identified as being positive for bone metastasis (BSI of 0.4%) as the area of increased uptake is in a large urinary bladder (as marked in red) and not in a metastatic lesion. BSI = bone scan index.

**Figure 9:**
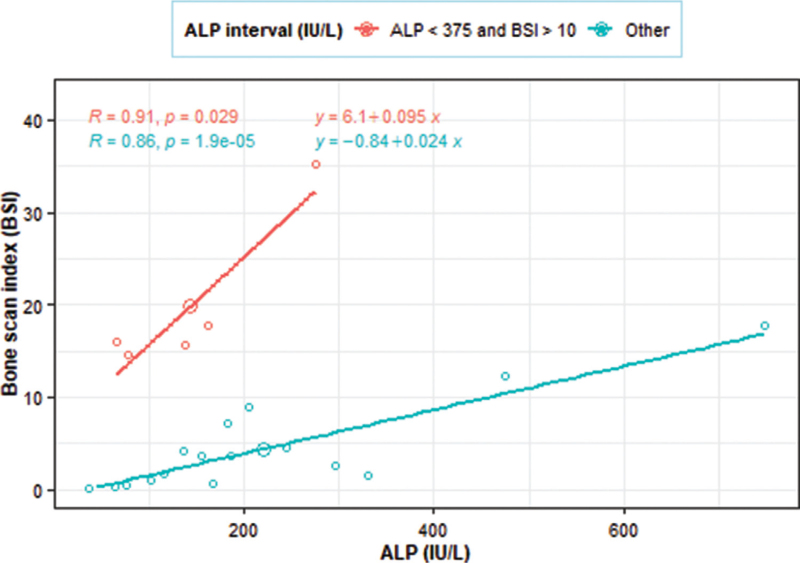
The anterior and posterior images to the left are the original bone scan images and the anterior and posterior images to the right are the processed BSI images. In this patient (patient 44), the BSI was calculated as 3.7%; however, this was an overestimation of the BSI as numerous degenerative and inflammatory lesions were also identified as areas of metastases incorrectly. BSI = bone scan index.

The automated BSI correlated correctly with 46/50 (92%) of the bone scans. In 2 (4%) of the scans, the BSI correctly identified the cases as positive for bone metastases (BSI > 0); however, it grossly underestimated the extent of the metastases (Figs. 6 and 7). In one case (2%), the BSI was incorrectly identified as being positive for bone metastasis (Fig. 8), however, the increased uptake was in a large urinary bladder and not a metastatic lesion. In another case (2%), the BSI was calculated as 3.7% (Fig. 9); however, this was an overestimation of the BSI as numerous degenerative and inflammatory lesions were also identified as areas of metastases incorrectly.

### 3.8 Ability of PSA, ALP, and age to predict bone metastases

The sensitivity and specificity results in our study (Table 3) showed the potential of PSA, ALP, and age as diagnostic tools for prostate cancer metastasis. All 3 markers showed the same prevalence of 44% similar to the BSI findings. However, their diagnostic performances differed in some other aspects, such as false positives. The results revealed that using PSA ≥ 92.672 as an optimal cut-off point for detecting metastases, resulted in a diagnostic accuracy of 78% (specificity = 0.929, sensitivity = 0.591, and AUC = 0.874). Of the patients with PSA ≥ 92.672 that were classified as metastasis positive, 59.1% were genuinely positive for bone metastases. Similarly, of the patients with PSA <92.672 that were classified as metastasis negative, 92.9% were genuinely metastasis negative. The optimal cut-off point of PSA = 92.672 was found to correctly classify up to a maximum of 51.9% (Youden index) of bone scans without a false positive. The ALP = 129.528 optimal cut-off point has been shown to have an effectiveness of 57.5%, that is, the maximum attainable sensitivity without a false positive. The diagnostic accuracy of ALP = 129.528 optimal cut-off point was 80%, and could genuinely detect 68.2% (sensitivity) of the bones as metastasis positive and 89.3% (specificity) as genuinely metastasis negative. Both PSA and ALP optimal cut-off points showed areas under the curve (AUC) of 0.874 and 0.808, respectively, an indication that they were useful tests for discriminating negative and positive metastasis cases. Age was not promising in terms of metastasis detection with an effectiveness of just 15.3% (Youden index) and an AUC of 0.636.

**Table 3:** Comparison between PSA, ALP, and age in predicting metastatic spread demonstrating the optimal cut-off point for detecting metastases when using these parameters.

Predictor	Metastasis optimal cut-off point	Youden index	Diagnostic accuracy	Sensitivity	Specificity	AUC	Prevalence
PSA	≥92.672	0.519	0.780	0.591	0.929	0.874	0.440
ALP	≥129.528	0.575	0.800	0.682	0.893	0.808	0.440
Age	≥67.691	0.153	0.580	0.545	0.607	0.636	0.440

ALP = alkaline phosphatase, AUC = area under the curve, PSA = prostate specific antigen.

## 4 Discussion

The prevalence of prostate cancer has been increasing in recent years.^[1]^ One of the most frequent sites of metastasis from prostate cancer is bone. Detecting bone metastases is essential in predicting prognosis, and identifying or preventing complications incurred by disease progression.^[18]^ Bone metastases are present in up to 14% of patients at presentation and in 80% to 85% of those who die of the disease, and they therefore affect morbidity, reflect prognosis, and significantly influence decisions with regard to patient management.^[19]^

Ga-68 PSMA PET/CT is superior to a bone scan for staging/restaging prostate cancer patients as it is has a higher sensitivity for the detection of bone metastases as well as it is able to detect extraosseous metastases. According to a study carried out by Caglar et al,^[20]^ they demonstrated that 75% and 98.2% of patients with bone metastases were correctly diagnosed by bone scintigraphy and Ga-68 PSMA PET/CT, respectively. It was noted that although Ga-68 PSMA PET/CT has better image resolution and diagnostic confidence than bone scintigraphy, it does comes at a higher cost.^[20]^ Due to the cost of the camera and the radiopharmaceutical being high, Ga-68 PSMA PET/CT is therefore still not widely available and only limited to a few centers in South Africa and many low income countries, thus many centers still rely on a bone scan as part of the staging/restaging work-up.

The BSI makes it possible to quantify bone involvement as well as therapeutic response. It was originally developed by Imbriaco et al^[6]^ but BSI has not become widely accepted because it has been found to be time-consuming (requires 20–40 minutes per scan) and requires special training to apply it to routine clinical work.^[21,22]^ The BSI appeared in the late 1990s, originating from the Memorial Sloan Kettering Cancer Centre.^[21]^ It provided quantification of bone metastases based on an algorithm that incorporated the weight of each bone expressed as the fraction of weight of the entire skeleton and the fractional involvement of each bone by tumor estimated from the whole-body bone scan.^[23]^ The output was the BSI, which expressed the percentage of involved bone as a percentage of the entire skeleton.^[23]^

Computer-assisted diagnosis systems, which are an automated platform for BSI, have been developed and are now available to address the problems of calculating the BSI manually. One of the problems was that it was time-consuming; however, with the computer automation, the time of detecting metastatic lesion/s and calculating the BSI is reduced to 3–5 seconds per scan.^[22]^ Kaboteh et al,^[24]^ showed that the automated method had a sensitivity of 93% and a specificity of 87%. Since a computer-assisted diagnosis system can easily quantify bone scan image findings, those can be converted into a BSI in a more comprehensive and objective way to compare images obtained at different time points during the clinical course.^[21]^

In our study, the automated BSI correlated correctly with 46/50 (92%) of the bone scans. In 2 (4%) of the scans, the BSI correctly identified the cases as positive for bone metastases (BSI > 0), however, it grossly underestimated the extent of the metastases (Fig. 6—patient 30 had a PSA of 1366.09, ALP of 167, and a BSI of 0.6%, and Fig. 7—patient 46 had a PSA of 460.61, ALP of 1487, and a BSI of 0.1%). These were “superscans” on visual assessment, which indicate widespread osteoblastic skeletal metastases and should thus have been allocated much higher bone scan indices. The BSI is calculated as the sum of the fractions of the skeleton with metastatic hot foci. As these scans had coalescing areas of bone metastases, the computer programme was unable to fractionate these areas adequately, and this thus resulted in an underestimation of the BSI. Thus one of the drawbacks of the automated BSI that we found in our study was in the case of “superscans.” This is similar to the findings of Ulmert et al,^[25]^ who found that the automated BSI method tended to underestimate BSI scores in patients with extensive disease.

In one case (2%), the BSI was identified as being positive for bone metastasis (Fig. 8—patient 42 had a BSI of 0.4%) as indicated by a large area of increased uptake in the pelvis. However, on visual inspection, this was actually a large urinary bladder and not a metastatic lesion. The BSI was then changed to 0% (negative for bone metastasis). Manual corrections for obvious misclassifications of bone lesions is performed from time to time as was also undertaken in the study by Armstrong et al.^[26]^ In this study, the automated software incorrectly classified 3 cases as having bone lesions when the uptake present was actually due to radiotracer activity in the bladder.^[26]^ The study by Ulmert et al^[25]^ shows other instances where manual corrections needed to be carried out for urine contamination and for a urinary catheter attached to a drainage bag.

In another case (2%), the BSI was calculated as 3.7% (Fig. 9—patient 44); however, on visual examination, numerous degenerative and inflammatory lesions were also marked as areas of metastases incorrectly. This thus resulted in an overestimation of the BSI. Hotspots in a bone scan are non-specific,^[24]^ and false positives for metastatic lesions may be seen in degenerative changes, inflammation, and trauma.^[21]^ The computer programme is able to differentiate metastatic lesions from degenerative changes. However, there are instances where it will not reliably do so as in our study.

Despite the above-mentioned limitations of the automated BSI, it is still quite accurate and precise, and it remains an excellent tool for quantifying bone metastases as it provides a reproducible method.

In terms of demographics, our study showed that there was no statistically significant difference in the median ages between the positive and negative patients for bone metastases. This is similar to the findings of Lin et al,^[27]^ who found no difference in age between those patients with bone metastases and those without. Our study also showed that the median PSA was very high among Blacks as compared with the PSA medians of Indians and Whites. This is similar to the findings of the study carried out by Sathekge et al^[28]^ who found that serum PSA values in black South Africans was significantly higher when compared with White South Africans. According to Sathekge et al,^[28]^ this finding reflects the well-documented higher known disease burden found in Black patients at initial staging. In addition, androgen receptor mutations and polymorphisms regulating PSA production as well as PSA gene polymorphisms between black and white South Africans may also contribute to this finding.^[28]^ In our study, we also found that the median ALP was the highest for Indians as compared with the ALP medians of Blacks (moderate) and Whites (lowest). Unfortunately, it was not possible to correlate this finding to other studies due to the lack of literature available regarding this.

The purpose of our study was to compare the BSI correlation to PSA against that of BSI to ALP levels in patients with a Gleason score ≥7. Our study showed that the optimal cut-off point of PSA = 92.672 was found to correctly classify up to a maximum of 51.9% (Youden index) of bone scans without a false positive. Numerous studies around the world indicate that PSA > 20 ng/mL is more accurate in predicting metastasis on bone scan.^[29]^ Armstrong et al^[26]^ highly recommend that at PSA level >24 ng/mL, a bone scan should be performed as PSA level of 24 ng/mL indicates a 63% chance of a positive bone scan. Similarly, Manohar et al^[30]^ found that the optimal cut-off value of PSA for positive skeletal metastasis is >29.16 ng/mL, and the chances of getting a positive bone scan for skeletal metastasis are less in patients with PSA <29.16 ng/mL. The optimal cut-off point of PSA in our study was much higher at 92.672. One of the reasons for this may be that different regions of the world suggest different cut-off values of PSA levels in the prediction of skeletal metastases on bone scan.^[30]^ Although the study by Omar et al^[29]^ was carried out in South Africa with a large number of Blacks making up the study population (72.62%) which is similar to ours (50%), it was, however, carried out in a different locality to ours. This suggests that even different regions in the same country may have different cut-off values of PSA, and that this value may actually be site-specific. Another reason for the difference in cut-off values of PSA is that the PSA values in our study ranged from 0.0400 to 2790 ng/mL whereas in the study by Omar et al,^[29]^ PSA values >100 were classed together and it is uncertain how high these values actually went. If our study did in fact, have higher PSA values than their study, this would explain the higher PSA cut-off values.

For the metastasis positive measurements, there was a statistically significant moderate positive overall linear correlation (*r* = 0.54, *P* = .009) between PSA and BSI. For ALP and BSI, there were 2 segmented strong positive linear relationships between them. The first segment consisted of ALP <375 IU/L and BSI >10%, where ALP and BSI were strongly and positively correlated (*r* = 0.91, *P* = .029). The other segment tended to have generally low BSI measurements (<10%) and also had a strong and positive correlation (*r* = 0.86, *P* < .001). These findings indicate that BSI correlates better with ALP than PSA. This is similar to the findings of the study carried out by Wymenga et al^[7]^ who found that ALP levels correlated better than PSA levels with bone scan results.

Of the 22 metastasis positive patients, 40.9% (9/22) had their ALP measurements obtained >6 months prior to the bone scan. In our study population, PSA levels were checked routinely in patients as it is a tumor marker that is used to monitor treatment response and detect recurrence. Unfortunately, ALP levels were not routinely monitored. Our study has shown that the BSI correlates better with ALP than PSA, and thus, we hope that, going forward, ALP levels will be monitored more frequently in prostate cancer patients.

There are a minimal number of studies assessing the BSI correlation to PSA against that of BSI to ALP, or even just assessing the BSI correlation to ALP. Also, when collecting data for our study, we found that ALP levels were not checked as often as PSA levels, hence some of the results being from more than six months prior to the bone scan. Since our study has shown the importance of ALP levels, it is imperative that clinicians should be checking ALP levels more frequently. In addition, more studies are required to assess the BSI correlation to ALP.

The limitations of our study was a relatively small sample size as well as the retrospective nature, which may have resulted in selection bias because a lot of the patients did not have an ALP result and were therefore excluded. Further larger studies are recommended to confirm the findings in our study.

## 5 Conclusion

BSI was found to be better correlated with ALP than PSA, and the relationship was clearly defined in sub-segments rather than from an overall view. Both ALP and PSA can potentially be used to predict bone metastases and to decide which patients should be referred for bone scintigraphy in resource-limited settings. Our study has shown the importance of ALP levels in assessing for metastases, and thus further studies should be carried out to confirm this.

## Author contributions

**Conceptualization:** Nozipho Nyakale, Tasmeera Ebrahim.

**Data curation:** Tasmeera Ebrahim.

**Formal analysis:** Partson Tinwaro.

**Investigation:** Tasmeera Ebrahim.

**Methodology:** Bawinile Hadebe, Colleen Aldous, Nozipho Nyakale, Tasmeera Ebrahim.

**Project administration:** Bawinile Hadebe, Tasmeera Ebrahim.

**Software:** Bawinile Hadebe.

**Supervision:** Nozipho Nyakale.

**Validation:** Colleen Aldous, Nozipho Nyakale.

**Visualization:** Colleen Aldous, Nozipho Nyakale, Tasmeera Ebrahim.

**Writing – original draft:** Tasmeera Ebrahim.

**Writing – review & editing:** Bawinile Hadebe, Colleen Aldous, Nozipho Nyakale, Partson Tinwaro, Tasmeera Ebrahim.
